# HNPCC *versus *sporadic microsatellite-unstable colon cancers follow different routes toward loss of HLA class I expression

**DOI:** 10.1186/1471-2407-7-33

**Published:** 2007-02-22

**Authors:** Jan Willem F Dierssen, Noel FCC de Miranda, Soldano Ferrone, Marjo van Puijenbroek, Cees J Cornelisse, Gert Jan Fleuren, Tom van Wezel, Hans Morreau

**Affiliations:** 1 Department of Pathology, Leiden University Medical Center, Leiden, The Netherlands; 2 Department of Immunology, Roswell Park Cancer Institute, Buffalo, NY, USA

## Abstract

**Background:**

Abnormalities in Human Leukocyte Antigen (HLA) class I expression are common in colorectal cancer. Since HLA expression is required to activate tumor antigen-specific cytotoxic T-lymphocytes (CTL), HLA class I abnormalities represent a mechanism by which tumors circumvent immune surveillance. Tumors with high microsatellite instability (MSI-H) are believed to face strong selective pressure to evade CTL activity since they produce large amounts of immunogenic peptides. Previous studies identified the prevalence of HLA class I alterations in MSI-H tumors. However, those reports did not compare the frequency of alterations between hereditary and sporadic MSI-H tumors neither the mechanisms that led to HLA class I alterations in each subgroup.

**Methods:**

To characterize the HLA class I expression among sporadic MSI-H and microsatellite-stable (MSS) tumors, and HNPCC tumors we compared immunohistochemically the expression of HLA class I, β2-microglobulin (β2m), and Antigen Processing Machinery (APM) components in 81 right-sided sporadic and 75 HNPCC tumors. Moreover, we investigated the genetic basis for these changes.

**Results:**

HLA class I loss was seen more frequently in MSI-H tumors than in MSS tumors (p < 0.0001). Distinct mechanisms were responsible for HLA class I loss in HNPCC and sporadic MSI-H tumors. Loss of HLA class I expression was associated with β2m loss in HNPCC tumors, but was correlated with APM component defects in sporadic MSI-H tumors (p < 0.0001). In about half of the cases, loss of expression of HLA class I was concordant with the detection of one or more mutations in the *β2m *and APM components genes.

**Conclusion:**

HLA class I aberrations are found at varying frequencies in different colorectal tumor types and are caused by distinct genetic mechanisms. Chiefly, sporadic and hereditary MSI-H tumors follow different routes toward HLA class I loss of expression supporting the idea that these tumors follow different evolutionary pathways in tumorigenesis. The resulting variation in immune escape mechanisms may have repercussions in tumor progression and behavior.

## Background

During cancer development, tumor cells may elicit cytotoxic T-lymphocyte (CTL)-mediated immune responses–partly a consequence of accumulated gene mutations that are translated into altered peptides [1]. Tumor cell expression of HLA class I-antigen complexes is essential for CTL recognition of aberrant peptides and subsequent activation [2]. Consequently, alteration of HLA class I cell surface expression provides an effective mechanism by which tumors can escape immune detection [3,4]. Multiple mechanisms have been shown to underlie defects in HLA class I expression by tumor cells. They include mutations in the individual HLA class I genes *HLA-A*, *-B *and *-C*, located on chromosome 6p21.3) [5]; mutations in *β2-microglobulin *(*β2m*) [6-9], molecule required for cell surface expression of HLA class I antigens; and defects in components of the HLA class I-associated antigen-processing machinery (APM) [9-11]. The APM consists of proteasome components delta, MB1 and Z; the immunoproteasome components LMP2, LMP7 and LMP10; peptide transporters TAP1 and TAP2; and chaperones Calnexin, Calreticulin, ERp57, and Tapasin. The immunoproteasome generates peptides mostly, although not exclusively from endogenous proteins, TAP1 and TAP2 facilitate peptide translocation from the cytosol into the lumen of the endoplasmic reticulum, where the peptides are loaded onto the HLA class I molecules with the aid from the several chaperones [12].

Chromosomal instability (CIN) and microsatellite instability (MIN) are the two major forms of genetic instability in colorectal cancer. Combined with distinct somatic mutation patterns and epigenetic modifications, CIN and MIN lead to the development of sporadic colorectal cancer [13]. MIN sporadic tumors, which constitute approximately 15% of all colorectal cancer cases and up to 40% of the tumors localized on the right side (preceding the splenic flexure) of the colon [14], have a phenotype resulting from the epigenetic inactivation of the mismatch repair gene *hMLH1*. Its inactivation destroys a cell's ability to repair base-base mismatches and small insertions or deletions in repetitive stretches, leading to an accumulation of frameshift mutations that get translated into abnormal peptide sequences. When these mutations are accumulated to large extent in the cell genome the tumors are said to possess high-microsatellite instability (MSI-H) [15]. Hence, it is expected that genes containing microsatellite sequences within their coding regions are more susceptible to somatic mutations, as seen in the *TGFβ-RII *gene. *TGFβ-RII's *third exon contains a microsatellite repeat of 10 adenines that is frequently targeted by frameshift mutations in MSI-H tumors [16]. MSI-H is also the hallmark of hereditary non-polyposis colorectal cancer (HNPCC), in which germline mutations of *hMLH1*, *hMSH2*, *hMSH6 *and *PMS2 *can be found. HNPCC constitutes approximately 2–4% of all CRC cases [17]. Tumors with MSI-H are thought to be more able to stimulate a CTL-mediated immune response due to their frequent generation of the aberrant frameshift peptides [18]. Therefore, these tumors are subjected to a greater selective pressure which favors the outgrowth of tumor cells with the ability to escape from recognition and destruction by host immune system.

Various studies have identified HLA alterations in colorectal cancer [19-21], including the prevalence of HLA class I alterations in MSI-H tumors [8,22]. However, the latter studies did not compare the frequency of alterations between hereditary and sporadic MSI-H tumors neither the mechanisms that led to HLA class I alterations in each subgroup. It was suggested that MSI-H sporadic and hereditary tumors follow parallel evolutionary pathways during tumorigenesis in terms of both genotype and phenotype [23]. As far as HLA class I defects are concerned it was never investigated whether these different tumors present distinct escape mechanisms from the immune system. In the present study, we compared the frequency of defects in HLA class I expression in right-sided sporadic (MSI-H and microsatellite-stable (MSS) sub-groups) colon tumors and in HNPCC tumors and studied the mechanisms underlying any abnormalities in these subgroups.

## Methods

### Patient material and tissue microarrays

Two tissue microarrays were constructed from formalin-fixed, paraffin-embedded tissues as described previously [24]. One array, previously described [25], included colorectal tumor specimens from 129 suspected HNPCC patients with MSI-H colon tumors of which 75 cases were analyzed in the present study after confirmation of their HNPCC status: 73.3% (n = 55) of the latter possessed a germline pathogenic mutation in *hMLH1 *(n = 24), *hMSH2 *(n = 18), *hMSH6 *(n = 12) or *PMS2 *(n = 1), the remaining were MSI-H, without methylation of the *hMLH1 *promoter and/or with immunohistochemical loss of the MSH2/MSH6 heterodimer and/or possessed a very young age at diagnosis of colon cancer (<50 yrs old). All cases possessed a positive family history for MSI-H tumors. The second tissue array included 3 tumor tissue cores from 81 sporadic right-sided colon cancer cases resected between 1990 and 2005 at the Leiden University Medical Center (Leiden, The Netherlands) and at the Rijnland Hospital (Leiderdorp, The Netherlands). The 81 patients in the latter array consisted of 47 females and 34 males with a mean age of 71.15 years (SD= 9.958). Approximately 60% (n = 48) of these cases were classified as MSS while the remaining (n = 33) possessed a MSI-H phenotype. The microsatellite instability status of the tumors was determined according to recommendations of the National Cancer Institute/ICG-HNPCC [15]. Moreover all MSI-H sporadic cases have lost the expression of the MLH1/PMS2 heterodimer as assessed by immunohistochemistry. The sporadic status of the MSI-H right-sided tumors (RST) was confirmed by methylation analysis of the *hMLH1 *promoter using a methylation-specific MLPA assay as previously described [26]. All MSI-H sporadic cases presented with hypermethylation at the *hMLH1 *promoter.

The present study falls under approval by the Medical Ethical Committee of the LUMC (protocol P01–019). Cases were analyzed following the medical ethnical guidelines described in the Code Proper Secondary Use of Human Tissue established by the Dutch Federation of Medical Sciences [27].

### Immunohistochemistry

Standard three-step, indirect immunohistochemistry was performed on 4-μm tissue sections transferred to glass slides using a tape-transfer system (Instrumedics, Hackensack, NJ), including citrate antigen retrieval, blockage of endogenous peroxidase and endogenous avidin-binding activity, and di-aminobenzidine development.

The following primary antibodies were used: the mAb HCA2 which recognizes β2m-free HLA-A (except -A24), -B7301 and -G heavy chains [28,29] ; the mAb HC10, which recognizes a determinant expressed on all β2m-free HLA-B and C heavy chains and on β2m-free HLA-A10, -A28, -A29, -A30, -A31, -A32 and -A33 heavy chains (supernatant kindly provided by Dr. J. Neefjes, NKI, Amsterdam, The Netherlands and Dr. H. L. Ploegh, MIT, Boston, MA) [28,30]; TAP1 specific mAb NOB1; LMP2-specific mAb SY-1; LMP7-specific mAb HB2; LPM10-specific mAb TO-7; Calnexin-specific mAb TO-5; Calreticulin-specific mAb TO-11; Tapasin-specific mAb TO-3; ERp57-specific mAb TO-2 [31-33]; TAP2-specific mAb (BD Biosciences Pharmingen, San Diego, CA); rabbit anti-β2m polyclonal Ab (A 072; DAKO Cytomation, Glostrup, Denmark); anti-MLH1 (clone G168–728; BD Biosciences) and anti-PMS2 (clone A16-4; BD Biosciences). Secondary reagents used were biotinylated rabbit anti-mouse IgG antibodies (DAKO Cytomation), goat anti-rabbit IgG antibodies (DAKO Cytomation), and biotinylated-peroxidase streptavidin complex (SABC; DAKO Cytomation).

Loss of expression was defined by complete lack of staining in membrane and cytoplasm (HCA2, HC10, and anti-β2m), in the nucleus (anti-MLH1 and anti-PMS2), in the peri-nucleus/endoplasmic reticulum (NOB1, anti-TAP2, TO-2, TO-3, TO-5, TO-7, and TO-11), or in the cytoplasm (SY-1, HB2, and TO-7), but with concurrent staining in normal epithelium, stroma or infiltrating leukocytes. HLA class I expression was considered to be lost when one of the HLA class I antigen-specific antibodies gave a negative result alongside a positive internal control (lymphocytic infiltrate).

### Flow cytometric sorting

The flow cytometric sorting procedure, including tissue preparation, staining and flow cytometry analysis was performed as described previously [34]. Briefly, 2 mm diameter punches from selected areas of formalin-fixed paraffin embedded colorectal carcinomas were digested enzymatically in a mixture of 0.1% collagenase I-A (Sigma-Aldrich, St Louis, MO, USA) and 0.1% dispase (Gibco BRL, Paisley, UK). After determination of cell concentration, one million cells were incubated with 100 μl of mAb mixture directed against keratin and vimentin containing clones MNF116 (anti-keratin; IgG1; DAKOCytomation, Golstrup, Denmark), AE1/AE3 (anti-keratin; IgG1; Chemicon International Inc, Temecula, CA, USA), and V9-2b (anti-vimentin; IgG2b; Department of Pathology, LUMC [35]). Next day, cells were incubated with 100 μl of premixed FITC and RPE-labelled goat F(ab')2 anti-mouse subclass-specific secondary reagents (Southern Biotechnology Associates, Birmingham, AL, USA). After washing, cells were incubated with 10 μM propidium iodide (PI) and 0.1% DNase-free RNase (Sigma). The next day cells were analyzed by flow cytometry. A standard FACSCalibur (BD Biosciences) was used for the simultaneous measurement of FITC, RPE, and PI. Tumor and normal cell populations were flow-sorted using a FACSVantage flow-sorter (BD Biosciences) using the FACSCalibur filter settings. Sorting was only performed on samples included in the RST array due to shortage of material from the HNPCC cases. DNA from flow-sorted tumor material was isolated as described by Jordanova *et al*. [36]. DNA from non-sorted material was isolated using Chelex extraction as described previously [37].

### LOH and fragment analysis

Markers for loss of heterozygosity (LOH) analysis were chosen from the dbMHC database [38] to map the chromosome 6p21.3 region between HLA-A and TAP2. They were MOGc, D6S510, C125, C141, D6S2444, TAP1 and M2426. A "linker" sequence of 5'-GTTTCTT was added to the 5' terminus of all reverse primers [39]. LOH was defined as allelic imbalance >2 in the HNPCC cases (non-sorted) and allelic imbalance >5 in the sorted RST [40].

To detect frame-shift mutations in the *HLA-A*, *HLA-B*, *β2m, LMP2*, *LMP7*, *LMP10*, *TAP1*, *TAP2*, *Calnexin*, *Calreticulin*, *ERp57 *and *Tapasin *genes, 28 pairs of primers (Table 1) were constructed surrounding non-polymorphic microsatellite regions within the coding regions.

### Statistics

Significance values were calculated using the software package SPSS 10.0.7 (SPSS Inc., Chicago, IL, USA).

## Results

### HLA class I, β2m and APM component expression

**Figure 1:**
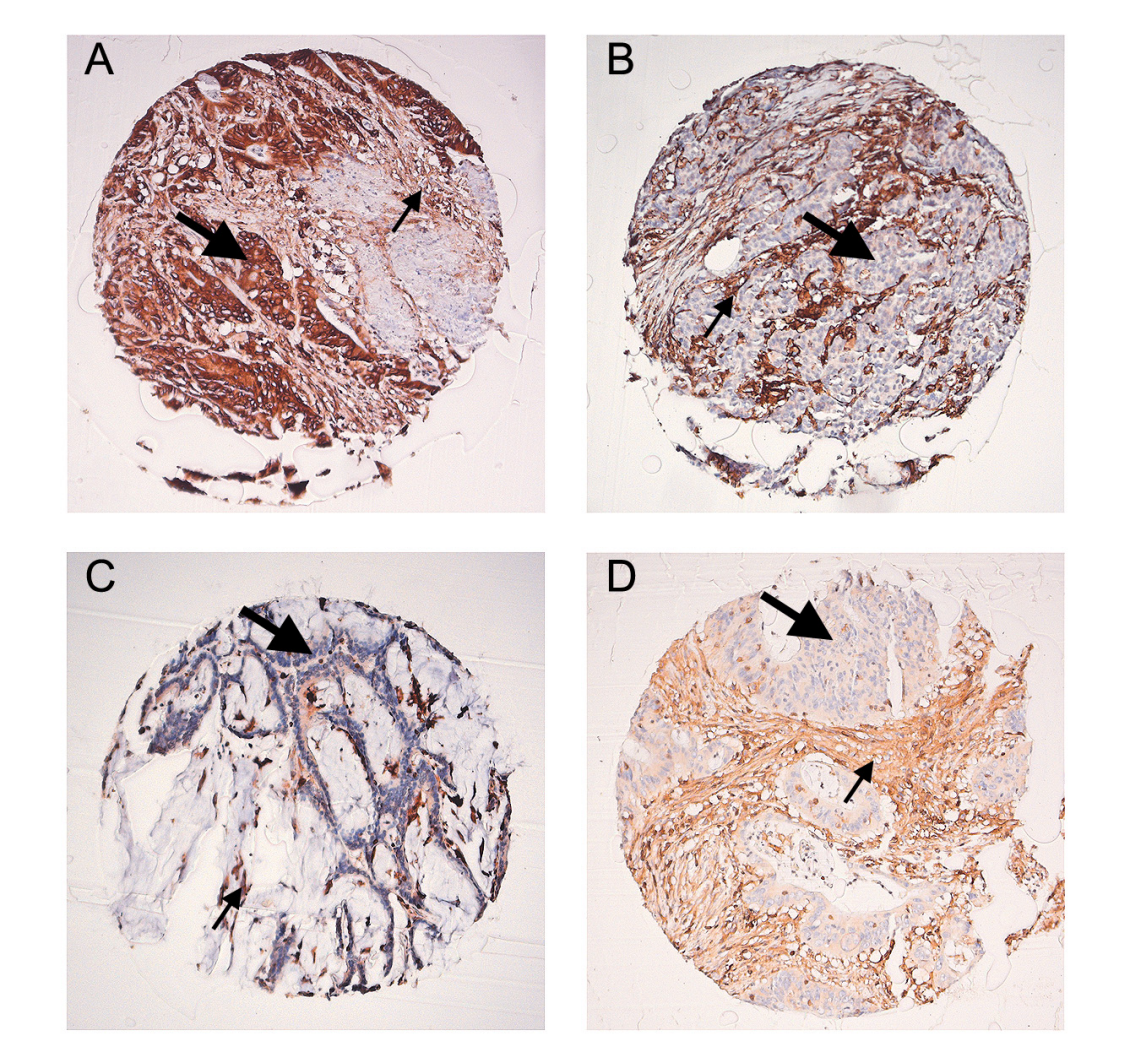
Example of immunohistochemical analysis performed on the RST and HNPCC tumors (Amplification 10×). A, Positive expression of HLA class I antigens detected with the HCA2 antibody. The epithelial (large arrow) membranous expression of HLA class I antigens is identical to the lymphocytic infiltrate (small arrow). B, Loss of expression of HLA class I identified with the HCA 2 antibody. The lymphocytic infiltrate (small arrow) was used as a positive control to determine the loss of expression on the epithelial cells. C, Loss of expression of β2m in a HNPCC case. D, Loss of expression of one of the APM members (Tapasin) in a RST case.

In order to compare the expression of HLA class I in sporadic MSI-H and MSS right sided tumors (RST) and HNPCC MSI-H cases, we used an antibody panel recognizing monomorphic determinants expressed on HLA class I heavy chains, β2m and APM components (Figure 1).

In total, we identified loss of HLA class I expression in about 34.6% of the RST and 42.7% of the HNPCC cases. The frequency of alterations differed significantly between the sporadic MSS and MSI-H RST. The lack of HLA class I expression was more frequent in MSI-H sporadic cases than in MSS cases (p < 0.0001), as it was 16.7% in the latter group, but 60.6% in the former (Table 2).

**Figure 2:**
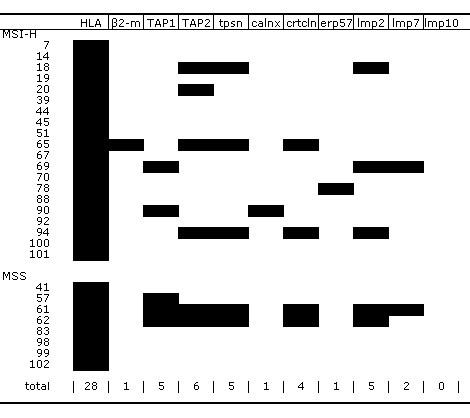
Loss of expression of β2m and different APM members was detected by immunohistochemistry in the RST that presented with HLA loss. The shadowing (in black) is indicative for loss of expression of the respective molecules. (tpsn – Tapasin, calnx – Calnexin, crtcln – Calreticulin)

Subsequently, we have investigated the frequency of a concomitant loss of HLA class I expression with that of either the β2m molecule or of any APM component. In the sporadic subset, loss of HLA class I expression was more often associated with that of one of the APM components, occurring in about 37% of HLA-negative tumors regardless of their mismatch repair status (Table 2). β2m loss was only found in one HLA class I negative MSI-H sporadic tumor (case 65) that interestingly also presented loss of the APM molecules TAP2, Calreticulin and Tapasin (Figure 2). In contrast, loss of HLA class I expression in HNPCC cases was more frequently associated with that of β2m (Table 2), as it was found in 46.9% of the HLA class I-negative tumors. In contrast loss of any APM component was observed in only 6% of these cases (h38, h49) which also showed loss of β2m expression.

In sporadic RST, the simultaneous loss of more than one APM molecule per case was frequent (Figure 2). Only 3 out of 10 cases lost a single APM component. The TAP2 molecule was most frequently lost (6 cases), followed by TAP1, Tapasin and LMP2 (5 cases), Calreticulin (4 cases), LMP7 (2 cases), and Calnexin and ERp57 (1 case). Loss of the LPM10 protein was detected in neither sporadic RST nor HNPCC tumors. The HNPCC cases h38 and h49 lost the expression of TAP2 and LMP7 respectively.

### LOH and frameshift analysis

**Figure 3:**
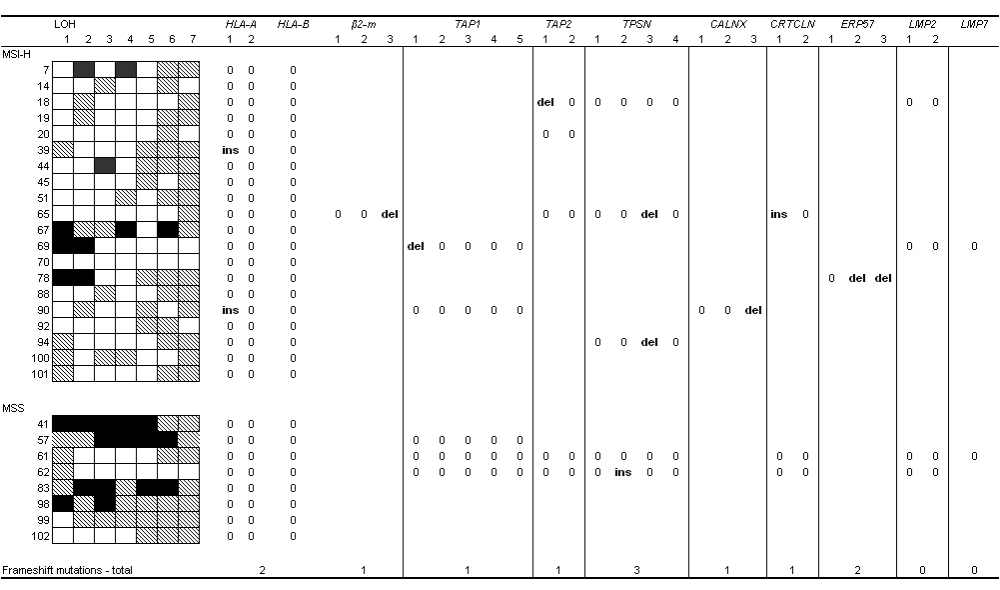
LOH and frameshift analysis was performed on sporadic RST that lost HLA class I expression. Only the tumors that presented with loss of one of the APM molecules or β2m were subjected to fragment analysis in their respective genes. The following repeats were analyzed for frameshift mutations: *HLA A*: 1 – 4^th ^exon 7(C), 2 – 5^th ^exon 3 (GGA); *HLA – B*: 3(GA) & 3(CA);*β2m*: 1 – 1^st ^exon 4(CT), 2 – 2^nd ^exon 4(GA) & 5(A), 3 – 2^nd ^exon 5(A); *TAP1*: 1 – 1^st ^exon 5(C), 2 – 3^rd ^exon 5(T), 3 – 8^th ^exon 5(G), 4 – 10^th ^exon 5(G), 5 – 11^th ^exon 6(G) & 5(A); *TAP2*: 1 – 2^nd ^exon 6(C), 2 – 9^th ^exon 5(G); *Tapasin*: 1 – 2^nd ^exon 5(G), 2 – 3^rd ^exon 5(C), 3 – 4^th ^exon 6(C), 4 – 5^th ^exon 5(G); *Calnexin*: 1 – 7^th ^exon 5(A), 2 – 8^th ^exon 5(A), 3 – 11^th ^exon 8(T); *Calreticulin*: 1 – 3^rd ^exon 5(G), 6^th ^exon 5(C); *ERp57*: 1 – 5^th ^exon 6(T), 2 – 6^th ^exon 6(A), 3 – 13^th ^exon 6(C); *LMP2*: 1 – 2^nd ^exon 5(G), 2 – 6^th ^exon 5(G); *LMP7*: 1^st ^exon 6(C) (key: ins – insertion; del – deletion; 0 – no mutation). LOH analysis of the 6p chromosome was also performed with the following markers: 1 – MOGc, 2 – D6S510, 3 – C125, 4 – C141, 5 – D6S2444, 6 – TAP1, 7 – M2426 (Key: Black – Loss of heterozygosity; Striped – non informative marker; White – Retention of heterozygosity).

**Figure 4:**
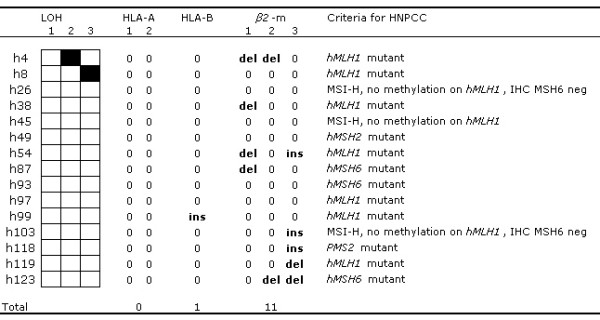
LOH and frameshift analysis was performed on HNPCC tumors that simultaneously lost HLA class I and β2m expression. LOH markers: (see legend from Figure 3 for key). Frameshift markers:*HLA A*: 1 – 4^th ^exon 7(C) 2 – 5^th ^exon 3 (GGA); *HLA – B*: 3(GA) & 3(CA); *β2m*: 1 – 1^st ^exon 4(CT), 2 – 2^nd ^exon 4(GA). & 5(A), 3 – 2^nd ^exon 5(A) (key: ins – insertion; del – deletion; 0 – no mutation; IHC -immunohistochemistry)

**Figure 5:**
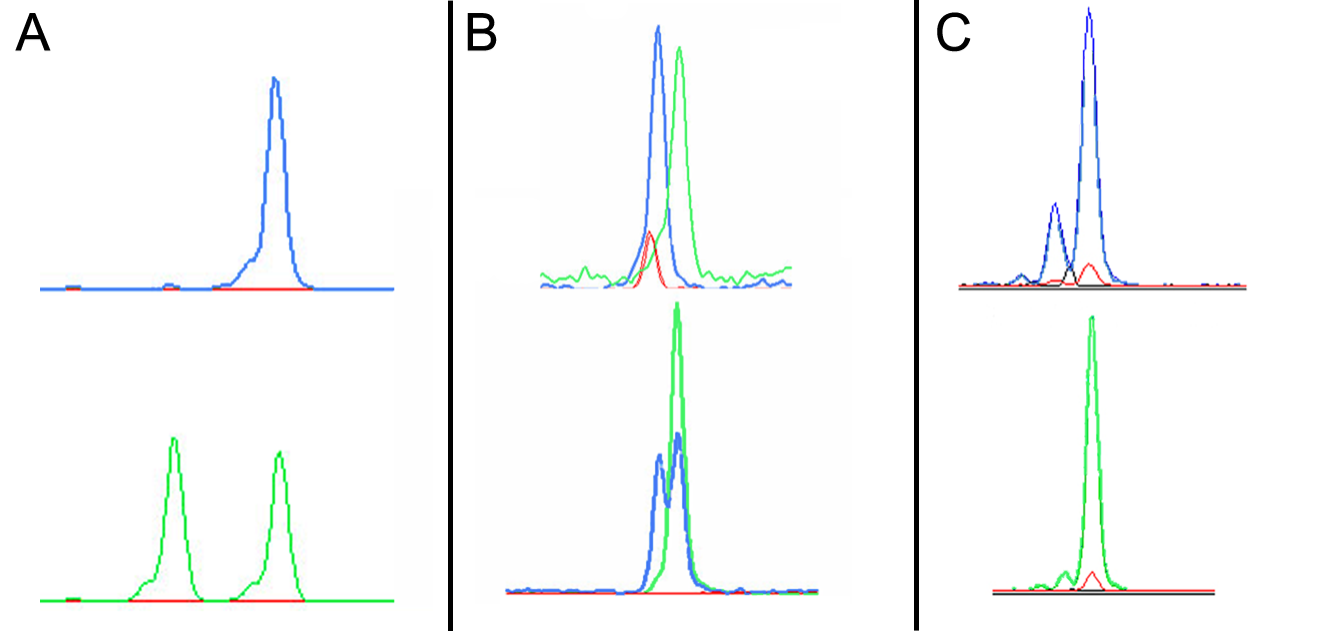
Genetic analysis performed on tumors that have lost HLA class I expression. The different peaks correspond to different sizes from the PCR-amplified products. Peaks corresponding to the normal samples are represented in green whereas tumor samples are represented in blue. A, LOH analysis performed on the RST 41 sample with the polymorphic marker C141. The total loss of a normal allele (on top) illustrates the technical advantage of using flow cytometric sorting to identify LOH events. B, Frameshift mutations identified in different members of the APM machinery. On top a homozygous deletion in the sample RST 65 on the 4^th ^exon of the *Tapasin *gene is shown. On the bottom, a heterozygous deletion in the sample RST 18 on the second exon of the *TAP2 *gene is shown. C, Frameshift mutation identified in one HNPCC case (h4) in the 2^nd ^exon of the *β2m *gene. Because flow sorting was not performed in the HNPCC cases, we cannot determine whether the frameshifts are homo- or heterozygous due to contamination with normal DNA.

Polymorphic markers around the classical *HLA *genes (*A*, *B *and *C*), *TAP 1 *and *TAP2 *genes were used to study LOH and reveal possible chromosomal aberrations that could relate to loss of HLA class I expression (Figure 5A). In HNPCC cases, LOH analysis was only performed around the HLA genes since loss of the *TAP1 *and *TAP2 *proteins was rarely associated with HLA class I loss. LOH was more frequent in the MSS tumors (50%) than in the MSI-H sporadic (20%) and HNPCC (6%) tumors with loss of HLA class I expression (P < 0.05) (Figure 3, 4). Furthermore, the patterns of LOH in the MSS cases might indicate loss of the entire 6p21.3 region, in contrast to the MSI-H cases (hereditary and sporadic forms) where LOH seems to be limited.

Frameshift mutation screening of the microsatellite sequences present in the coding regions of the *HLA class I*, APM components and *β2m *genes was performed on all cases with aberrant HLA class I expression (Figure 5B,C. However specific genes were only analyzed when lack of expression of the encoded proteins was detected by immunohistochemistry. Of the classical *HLA class I *genes only *HLA-A *and *-B *were analyzed since *HLA-C *does not carry any microsatellite repeat in its coding region. Ten RST cases and 20 HNPCC control cases with normal expression of β2m and APM components were screened for frameshift mutations and none was detected.

Frameshift mutations were mainly found in the MSI-H cases (both sporadic and hereditary forms). At least one mutation in an APM component gene was found in 6 of 7 sporadic MSI-H tumors that lost expression of one or more APM components. The single sporadic MSI-H case that lost β2m expression also presented with a single frameshift mutation in the *β2m *gene. Of the 13 sporadic MSI-H cases in which loss of HLA class I expression was associated neither with APM component nor with β2m loss as detected by immunohistochemical staining, one presented with a frameshift in an HLA gene (case 39, Figure 3, Table 3) while 3 other cases showed LOH of the markers adjacent the HLA genes (cases 7, 44 and 67). One frameshift mutation was found in the *Tapasin *gene in a MSS case (case 62). From 15 HNPCC tumors that lost β2m expression at least one mutation was found in 8 cases (Figure 4). Three of the latter showed 2 mutations localized in different stretches. In the remaining 17 HNPCC cases that solely lost HLA class I expression, only 4 showed genetic abnormalities. LOH was found in the HLA region in cases h16, h56 and h120 (data not shown). A frameshift mutation in one of the *HLA *genes (*HLA B*) was found in one case (h99, Figure 4). In neither of the 2 HNPCC cases that immunohistochemically lost the expression of one of the APM components an APM frameshift mutation was found.

## Discussion

Abnormalities in HLA class I cell surface expression are commonly observed in tumors and are interpreted as a mechanism by which tumor cells evade the host immune system [1]. In colorectal cancer, especially in MSI-H tumors, the high degree of lymphocytic infiltrate in some cases may suggest an active immune response during tumor development [41,42]. Moreover, MSI-H tumors might cause increased immune reactivity as a consequence of the high amounts of aberrant frameshift peptides they generate [8,18]. A selective pressure by CTLs upon these tumors would favor the outgrowth of tumor cells that lost HLA class I expression at the cell surface allowing them to surpass the action of the immune system.

Applying immunohistochemistry on tissue arrays, we compared HLA class I expression in both sporadic RST (MSI-H and MSS sub-groups) and HNPCC tumors. RST were chosen because of the high percentage of MSI-H cases in this specific tumor type [43]. Indeed, immunohistochemical staining with mAb showed that HLA class I loss was frequent in the MSI-H cases analyzed when compared to their MSS counterpart. This finding supports the hypothesis that MSI-H tumors face greater selective pressure to lose HLA class I expression, as described by Kloor *et al*[8]. However, we have shown for the first time that distinct molecular mechanisms underlie HLA class I loss in sporadic MSI-H and HNPCC colon cancers. In the latter, HLA class I loss was preferentially associated with that of β2m, while in the former HLA class I loss was associated with that of one or more APM components (p < 0.0001).

We investigated the genetic abnormalities underlying the HLA class I loss of expression. They included LOH on chromosome region 6p21.3 (encompassing *HLA *class I and *TAP *genes), mutations in APM components and mutations in *β2m*.

Loss of heterozygosity at 6p21.3 was most prevalent in MSS tumors. This is consistent with the observation that these tumors frequently possess gross chromosomal aberrations and are often aneuploid [13]. Moreover, since LOH events in MSS tumors normally comprise large areas of a chromosome, LOH on 6p21 might not be a direct consequence of selective pressure directed to the loss of HLA expression but instead to other genes within the same chromosomal region. The general absence of LOH in MSI-H tumors suggests that this is not the major mechanism by which the cells abrogate HLA class I expression.

The genome's coding regions contain multiple microsatellite repeats, which are considered hotspots for mutations in mismatch repair-deficient tumors [43]. Such repeats are also present within the exons of the APM components, *β2m*, *HLA-A *and *HLA-B *genes. In about half of the MSI-H cases, loss of expression of HLA class I was concordant with the detection of one or more mutations in these genes. We have discovered novel mutations in the antigen presenting machinery genes; *Tapasin*, *Erp57*, *Calreticulin *and *Calnexin *in colorectal cancer. Previous reports associated the loss of HLA class I expression in MSI-H tumors with defects on β2m molecule [7,9]. However, the authors did not distinguish the sporadic/hereditary nature of the tumors that were studied. We cannot exclude that the MSI-H cases included in these studies were mainly HNPCC tumors.

The reason sporadic MSI-H tumors would target APM members for inactivation and HNPCC would target the β2m chaperon is unclear. One possibility worth further exploration is that the various mutations suggest different immune-escape mechanisms for thwarting distinct anti-tumor responses. HNPCC tumors can have an age of onset before the 5^th ^decade of life while sporadic MSI-H tumors appear generally around the 7^th ^decade of life [43]; one would therefore predict that the alertness and robustness of the immune system would be higher in HNPCC patients leading to a stronger, or at least different selective pressure on the latter. Furthermore it has been recently suggested that the JC polyoma virus plays a role in the oncogenicity of colon tumors with an identical phenotype to sporadic MSI-H tumors [44]. Although speculative, the presence of the JC virus might be implicated in a different immune response between sporadic MSI-H and HNPCC tumors.

The advantages of different escape mechanisms (loss of APM members vs. abrogation of β2m) are not understood. The only known function of APM members is facilitating the expression of HLA classical molecules in complex with endogenous peptides. Thus, one would expect that only these HLA molecules would be affected by failure of the antigen processing machinery. On the other hand, it is accepted that cell surface expression of non-classical HLA molecules (e.g. HLA -G, -E) also depends on β2m, so the function of these highly specialized molecules would be compromised if β2m were mutated or lost. These molecules might play an important role in regulation of immune cell activity by inhibiting or activating its function. Therefore, MSI-H sporadic tumors that have lost expression of both HLA and an APM component and HNPCC tumors with lost β2m expression might behave differently or present a different kind of interaction with cells from the immune system. For instance, Yamamoto *et al*. have described a correlation between β2m mutations and unfavorable prognosis in colorectal cancer [45].

We separately analyzed the presence of the characteristic *BRAF *V600E somatic mutations in the RST cohort (data not shown). Forty-percent of MSI-H sporadic tumors presented with this mutation which was absent in the MSS tumors. It was previously described that this mutation is also absent in HNPCC tumors [46]. V600E was distributed equally between tumors that lost vs. retained expression of HLA class I in the sporadic MSI-H cases.

## Conclusion

HLA class I aberrations are found at varying frequencies in different colorectal tumor types and are caused by distinct genetic mechanisms. Chiefly, sporadic and hereditary MSI-H tumors follow different routes toward HLA class I loss of expression supporting the idea that these tumors follow different evolutionary pathways in tumorigenesis. The resulting variation in immune escape mechanisms may have repercussions in tumor progression and behavior.

## Competing interests

The author(s) declare that they have no competing interests.

## Authors' contributions

JWD – Contributed to the conception and design of the study, performed flow sorting procedure and was involved in the interpretation of data.

NM – Generated the RST array, performed the immunohistochemistry, flow sorting procedure, LOH and fragment analysis, was involved in the interpretation of data and drafting of the manuscript

SF – Contributed to the conception and design of the study, and critically reviewed the manuscript

MvP – Performed the MSI analysis on HNPCC cases and methylation-specific MLPA assay.

CC – Contributed to the conception and design of the study, and critically reviewed the manuscript

GJF – Contributed to the conception and design of the study, and to critical revision of the manuscript

TvW – Critically reviewed the manuscript.

HM – Contributed to the conception and design of the study, and is responsible for the study.

All authors read and approved the final manuscript.

## Pre-publication history

The pre-publication history for this paper can be accessed here:



## Figures and Tables

**Table 1:** Primers used in fragment analysis

Gene	Forward primer	Reverse primer	Microsatellite Repeat
*HLA A *4^th ^ex	CCTGAATTTTCTGACTCTTCCCGT	GTTTCTTTCCCGCTGCCAGGTCAGTGT	7(C)
*HLA A *5^th ^ex	CCATCGTGGGCATCATTG	GTTTCTTTCAGTGAGACAAGAAATCTC	3(GGA)
*HLA B *2^nd ^ex	GCTTCATCTCAGTGGGCTAC	GTTTCTTCTCGCTCTGGTTGTA	3(GA) + 3(CA)
*β2m *1^st ^ex	GGCTGGGCACGCGTTTAAT	GTTTCTTAGGGAGAGAAGGACCAGAG	4(CT)
*β2m *2^nd ^ex (1)	TACCCTGGCAATATTAATGTG	GTTTCTTGATAGAAAGACCAGTCCTTGC	4(GA) + 5(A)
*β2m *2^nd ^ex (2)	CTTACTGAAGAATGGAGAGAG	GTTTCTTGACTACTCATACACAACTTTCA	5(A)
*TAP1 *1^st ^ex	TAAATGGCTGAGCTTCTCGC	GTTTCTTAGAGCTAGCCATTGGCA	5(C)
*TAP1 *3^rd ^ex	ACAGCCACTTGCAGGGAG	GTTTCTTTATGAACAGTACATGGCGTAT	5(T)
*TAP1 *8^th ^ex	CTGCCCTGCTGCAGAATCTG	GTTTCTTCAAGCCACCTGCTTCCAT	5(G)
*TAP1 *10^th ^ex	CTCTGCAGAGGTAGACGAGG	GTTTCTTATTAAGAAGATGACTGCCTCAC	5(G)
*TAP1 *11^th ^ex	AGCACCTCAGCCTGGTGGA	GTTTCTTGCAGGTCTGAGAAGGCTTTC	6(G) + 5(A)
*TAP2 *2^nd ^ex	TTCCTCAAGGGCTGCCAGGAC	GTTTCTTGCTCCAAGGGGCTGAAG	6(C)
*TAP2 *9^th ^ex	CCTACGTCCTGGTGAGGTGA	GTTTCTTCTGGCTGTGCAGGTAGC	5(G)
*Tapasin *2^nd ^ex	TTGGTTCGTGGAGGATGC	GTTTCTTCCTAGAGACTCACCGTGTAC	5(G)
*Tapasin *3^rd ^ex	CTTCCTTCTCTACACTCAGACC	GTTTCTTAGGACTGGGCTGGATATGC	5(C)
*Tapasin *4^th ^ex	CCTGTCTTCCTCAGTGGTAC	GTTTCTTGAGCAGATGTCCCTTACCC	6(C)
*Tapasin *5^th ^ex	TGCTCATTTCGTCCTCTTTCC	GTTTCTTGTTCCCACTCCACCTCCAG	5(G)
Calnexin 7^th ^ex	GAAGGATCAGTTCCATGACAAG	GTTTCTTCTGCATCTGGCCTCTTAGC	5(A)
Calnexin 8^th ^ex	TCTGCTCAATGACATGACTCC	GTTTCTTTGAAGACAGTTCCCCAAGAC	5(A)
*Calnexin *11^th ^ex	AACCTTTCAGAATGACTCCTTTTAG	GTTTCTTCAAGCAGCAAACACGAACC	8(T)
Calreticulin 3^rd ^ex	CTACCGTCCCGTCTCAGG	GTTTCTTTCTGTCTGGTCCAAACTATTAGG	5(G)
Calreticulin 6^th ^ex	GACAAGCCCGAGCATATCC	GTTTCTTCACCTTGTACTCAGGGTTCTG	5(C)
*ERp57 *5^th ^ex	CACTTATTGCTTCTTCCTTGTG	GTTTCTTAATACTTGGTCAGGAGATTCAAC	6(T)
*ERp57 *6^th ^ex	CTTCTGCTATCTGCCTACTGAG	GTTTCTTTCAAGCAAATAAATCCCAGACAAG	6(A)
ERp57 13^th ^ex	ACTTTTAAGCTGATCTTTCTGTTTT	GTTTCTTTTAGAGATCCTCCTGTGCCTT	6(C)
*LMP2 *2^nd ^ex	GAGGGCATCAAGGCTGTTC	GTTTCTTGCAGACACTCGGGAATCAG	5(G)
*LMP2 *6^th ^ex	CCCTCTCTCCAACTTGAAACC	GTTTCTTTGTAATAGTGACCAGGTAGATGAC	5(G)
*LMP7 *1^st ^ex	GGCTTTCGCTTTCACTTCC	GTTTCTTGAGATCGCATAGAGAAACTGTAG	6(C)

**Table 2:** HLA, APM and β2m expression in RST and HNPCC colon cancer

		HLA^†^		APM loss ^‡^	β2m loss ^‡^
RST N = 81	MSS	+	40	0	0
	N = 48 (59,3%)	-	8 (16,7%)	3 (37,5%)*	0
					
	MSI-H	+	13	0	0
	N = 33 (41,7%)	-	20 (60,6%)	7 (35,0%)*	1 (5,0%)*

HNPCC N = 75	MSI-H	+	43	0	0
		-	32 (42,7%)	2 (6,3%)*	15 (46,9%)*

* – The percentage of cases that lost APM or β2m expression is relative to the number of HLA negative cases of each subset

† – HLA expression differs significantly between sporadic MSI-H and MSS tumors (p < 0.0001) and between HNPCC and sporadic MSS tumors (p < 0.005)

‡ – The association of HLA class I loss with that of β2m and of APM components differs significantly (p < 0.0001) between sporadic RST and HNPCC tumors.

**Table 3:** Frameshift mutations description in RST

RST	Mutation description
18	*TAP2 *(341–346)del1 – het
39	*HLA-A *(621–627)ins1 – het
65	*β2m *(341–345)del1 – hom; *Tapasin *(1217–1222)del1 – hom; *Calreticulin *(418–422)ins1 – hom
69	*TAP1 *(362–366)del1 – hom
78	*ERp57 *(675–680)del2 – hom; *ERp57 *(1459–1464)del1 – hom
90	*HLA-A *(621–627)ins1 – het; *Calnexin *(1476–1483)del1 – het
94	*Tapasin *(1217–1222)del1 – het

ins – insertion; del – deletion; hom – homozygous; het – heterozygous; the numbers before and after del/ins indicate the location of the microsatellite repeat containing the frameshift mutation and the number of affected nucleotides respectively.
